# Erratum

**DOI:** 10.4269/ajtmh.923err

**Published:** 2015-03-04

In *Am J Trop Med Hyg 90:* 1146–1152 by Carvalho do Espírito-Santo, the final version of this article should have contained the following statement: This study was supported by the São Paulo Research Foundation under processes 07/53457-7, 08/50461-6, 10/52615-0, and 14/04728-1. The journal regrets the error.

